# From Margin to Center: Intersectionality and the Politics of Environmental Gerontology

**DOI:** 10.1093/geroni/igab046.1537

**Published:** 2021-12-17

**Authors:** Austin Oswald, Jarmin Yeh, Jarmin Yeh

## Abstract

As the population becomes increasingly older and culturally diverse, so too does the need for critical scholarship that examines the complex lives of differently positioned older adults in relation to their social and physical ecologies. This innovative symposium reflects the importance of putting intersectional frameworks (Collins & Bilge, 2016) in conversation with environmental gerontology to critically examine structures of power in assessing who matters and who benefits from place-based initiatives that intend to support healthy aging (Phillipson, 2004). Perry et al.’s paper addresses the politics of responsibility, asking who is responsible for keeping older people safe in light of Covid-19 though a citywide senior housing coalition in Detroit. The second paper, authored by Johnson, speaks to the politics of access and structural inequalities that create disparities in end-of-life care for unhoused older adults. The third and fourth papers, by Stinchcombe and colleagues and Oswald, critically examine dominant paradigms of age-friendliness in Canada and the United States though a politics of representation that highlights who is (in)visible in these initiatives. The final paper by Reyes, on the civic participation of Latinx and African American older adults, illustrates how structural change cannot happen without engaging these populations in the political process. Together, these papers exemplify the politics of environmental gerontology and demonstrate that without acknowledgment of multi-layered identities and the structures contributing to their inequities, environmental gerontology is inadequate, as it may overlook important social and environmental factors that connect older people to the places where they live and die.

